# Apparent Splitting of S Waves Propagating Through an Isotropic Lowermost Mantle

**DOI:** 10.1002/2017JB014394

**Published:** 2018-05-11

**Authors:** Laura Parisi, Ana M. G. Ferreira, Jeroen Ritsema

**Affiliations:** ^1^ PSE Division King Abdullah University of Science and Technology Thuwal Saudi Arabia; ^2^ School of Environmental Sciences University of East Anglia Norwich UK; ^3^ Department of Earth Sciences University College London London UK; ^4^ CEris, ICIST Instituto Superior Técnico, Universidade de Lisboa Lisbon Portugal; ^5^ Department of Earth and Environmental Sciences University of Michigan Ann Arbor MI USA

**Keywords:** shear wave splitting, D' layer, mantle anisotropy, core‐mantle boundary, lowermost mantle

## Abstract

Observations of shear wave anisotropy are key for understanding the mineralogical structure and flow in the mantle. Several researchers have reported the presence of seismic anisotropy in the lowermost 150–250 km of the mantle (i.e., D ${}^{\prime \prime }$ layer), based on differences in the arrival times of vertically (*S* *V*) and horizontally (*S* *H*) polarized shear waves. By computing waveforms at a period > 6 s for a wide range of 1‐D and 3‐D Earth structures, we illustrate that a time shift (i.e., apparent splitting) between *S* *V* and *S* *H* may appear in purely isotropic simulations. This may be misinterpreted as shear wave anisotropy. For near‐surface earthquakes, apparent shear wave splitting can result from the interference of *S* with the surface reflection *s* *S*. For deep earthquakes, apparent splitting can be due to the *S* wave triplication in D ${}^{\prime \prime }$, reflections off discontinuities in the upper mantle, and 3‐D heterogeneity. The wave effects due to anomalous isotropic structure may not be easily distinguished from purely anisotropic effects if the analysis does not involve full waveform simulations.

## Key Points

- Simulations of wave propagation through the lowermost mantle display apparent *S* *H*‐*S* *V* splitting for 1‐D and 3‐D isotropic Earth models
- The apparent splitting is due to phase interference between *S* and reflected waves
- Anomalous Vs gradients, discontinuities, and heterogeneities may lead to misinterpretation of wave splitting

## 1 Introduction

The D ${}^{\prime \prime }$ layer—the lowermost 150–250 km of the mantle (Bullen, 1950)— plays a key role in global dynamics (for a recent review see, e.g., Lay, 2015). D ${}^{\prime \prime }$ is heterogeneous at various scales. It is characterized by anomalous radial wave speed gradients (e.g., Young & Lay, 1987a), a seismic discontinuity at its top (e.g., Lay & Helmberger, 1983; Wysession et al., 1998), large low‐shear velocity provinces (LLSVPs; e.g., Garnero et al., 2016; Lekic et al., 2012), ultralow‐velocity zones (e.g., Cottaar & Romanowicz, 2012; Garnero et al., 1993; Thorne et al., 2013), and anisotropic shear wave speed structure (e.g., Meade et al., 1995; Montagner & Kennett, 1996; Nowacki et al., 2011).

The presence of shear wave anisotropy, in particular, is important for interpreting the mineralogy and deformation of the D ${}^{\prime \prime }$ layer. Seismic anisotropy could be due to lattice‐preferred orientation of minerals (e.g., McNamara et al., 2002) such as postperovskite (e.g., Iitaka et al., 2004; Murakami et al., 2004; Oganov et al., 2005) or shape‐preferred orientation involving structural elements, such as layers of melt (e.g., Kendall & Silver, 1996). Possibly, deformation of ancient slabs which have subducted into the lowermost mantle may be responsible for the anisotropy (e.g., McNamara et al., 2002).

Seismic anisotropy in D ${}^{\prime \prime }$ is quantified by the difference in the arrival times or phase shifts of vertically (*S* *V*) and horizontally (*S* *H*) polarized shear wave phases (i.e., shear wave splitting) such as *S*, *S* *c* *S*, and Sdiff. Shear wave splitting up to 5 s, as reported in numerous studies, corresponds to radial anisotropy $\xi =V_{\text{SH}}^{2}/V_{\text{SV}}^{2}$ up to 1.06, depending on epicentral distance (Figure S1 in the supporting information). Most observations of anisotropy suggest that *V*_SH_ is higher than *V*_SV_ in regions of D ${}^{\prime \prime }$ where the shear velocity is relatively high (see Nowacki et al., 2011, for a recent review). These include the D ${}^{\prime \prime }$ region beneath Alaska (e.g., Garnero & Lay, 1997; Wysession et al., 1999), the Caribbean (Kendall & Silver, 1996), the Indian Ocean (Ritsema, 2000), and Siberia (Thomas & Kendall, 2002). The pattern of anisotropy is more complex within the LLSVPs and the transition zones between LLSVPs and the high‐velocity regions of D ${}^{\prime \prime }$. Here shear wave anisotropy is weak and recordings for similar source‐receiver paths provide evidence for *V*_SV_<*V*_SH_ and *V*_SV_>*V*_SH_ and azimuthal variations (e.g., Fouch et al., 2001; Garnero et al., 2004; Kendall & Silver, 1998; Pulliam & Sen, 1998; Ritsema et al., 1998; Vinnik et al., 1998, 1995).

It is not straightforward to interpret shear wave splitting and to construct models of anisotropy. Recent studies have shown that it is difficult to constrain D ${}^{\prime \prime }$ anisotropy using global tomographic inversions because *S* waves traveling in D ${}^{\prime \prime }$ are mostly sensitive to *V*_SH_. The unbalanced sensitivity to *V*_SH_ and *V*_SV_ results in leakage of heterogeneity into artificial anisotropic structure in D ${}^{\prime \prime }$ (e.g., Chang et al., 2014, 2015; Kustowski et al., 2008). Moreover, measuring shear wave splitting can be difficult because teleseismic *S* waves have low amplitudes after they have diffracted around the core (e.g., Doornbos & Mondt, 1979). Core diffraction and the interference with reflections off the core or layers within D ${}^{\prime \prime }$ affect *S* *V* and *S* *H* differently. Here we refer to the traveltime difference between *S* *H* and *S* *V* waves as “apparent splitting” when it is not due to seismic anisotropy.

The forward modeling tests by Borgeaud et al. (2016), Komatitsch et al. (2010), and Maupin (1994) demonstrate that the traveltimes of diffracted *S* *H* and *S* *V* waves can be different, even when the lowermost mantle has an isotropic shear wave structure. Maupin (1994) showed that the traveltime difference between *S* *H* and *S* *V* waves is not a discriminating factor between isotropic and anisotropic D ${}^{\prime \prime }$ models. She argued that particle motion can be used to constrain azimuthal anisotropy. Komatitsch et al. (2010) used spectral‐element method simulations for an earthquake at the Earth's surface to argue that the splitting between SHdiff and SVdiff can be as high as 15 s for 1‐D isotropic Earth models. Borgeaud et al. (2016) investigated the bias introduced by ray theory in the measurement of splitting in *S* waves traveling through the lowermost mantle and argued that *S* *H* and *S* *V* traveltimes can differ by as much as 16 s for 1‐D Earth models based on mineral physics and geodynamical information.

In this work, we expand on previous studies by investigating the effect of the earthquake source depth on waveforms and by exploring apparent splitting for a wide range of 1‐D and 3‐D isotropic structures for waveforms at periods longer than about 6 s. We study how wave interference affects the waveforms of *S* *H* and *S* *V* that propagate through D ${}^{\prime \prime }$, notably by analyzing *S*‐*s* *S* interference for shallow earthquakes and the *S* wave triplication at the top of D ${}^{\prime \prime }$ for deep earthquakes. We quantify apparent splitting for a large number of shear velocity models built by systematically (i) varying the thickness and radial shear velocity gradient in D ${}^{\prime \prime }$; (ii) considering a shear velocity discontinuity at the top of D ${}^{\prime \prime }$; and (iii) including large‐scale 3‐D shear velocity variations in the mantle.

## 2 Full Waveform Simulations of Deep Mantle Shear Waves

We compute synthetic seismograms using Gemini (GEM) (Friederich & Dalkolmo, 1995) and the spectral element method (SEM) (e.g., Komatitsch & Vilotte, 1998). Simulations based on 1‐D Earth models are run with 33 GEM and the simulations based on 3‐D Earth models are run with SEM. GEM is based on a minor integration technique and enables fast accurate waveform calculations at high frequencies and for 1‐D Earth models. GEM synthetics are calculated on a single processor core at maximum frequency of 200 mHz. GEM calculations use spherical harmonics up to degree 5,000 with a step of 1. On the other hand, SEM allows for the computation of waveform propagation through fully 3‐D Earth models (e.g., Komatitsch & Tromp, 2002a; Parisi & Ferreira, 2016; Parisi et al., 2015), but the simulations at short periods rely on fine meshes and relatively small time steps. We use the SPECFEM3D_GLOBE package (e.g., Komatitsch & Tromp, 2002a, 2002b) adapted for simulations to wave periods as short as 5.6 s and run simulations on 3,456 processor cores by splitting the mesh into 24 × 24 slices for each of the six chunks in which the globe is subdivided. The number of the elements at the surface of each chunk is set to 768 × 768. The length of the seismograms obtained from GEM and SEM simulations is 33 min.

We estimate time shifts (i.e., splitting) between *S* *V* and *S* *H* by manually identifying *S* *H* and *S* *V* onsets (*t*_SV_−*t*_SH_). For completeness, we also measure *t*_SV_−*t*_SH_ by using a cross‐correlation approach. Although cross–correlation measurements are more objective, they may be problematic in cases of waveform dissimilarity and differences in frequency content (Borgeaud et al., 2016). Overall, our splitting measurements obtained by cross correlation and from onsets are consistent when the cross correlation between the *S* *H* and *S* *V* waveforms is higher than 0.85 (see Figure S2 in the supporting information). Throughout this manuscript we discuss results based on onset measurements when the *S* phase onset can be clearly identified and there is good similarity between the *S* *H* and *S* *V* waveforms. Onset picks are not shown for sets of waveforms calculated with an Earth's model for which apparent splitting is not observed.

## 3 Apparent Splitting for a Shallow Earthquake Source

### 3.1 Method's Validation

**Figure 1:**
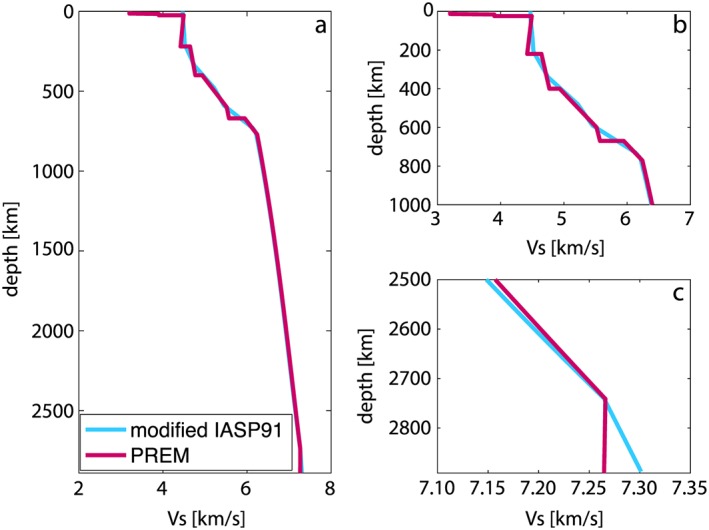
(a) Vs crustal and mantle profiles for the modified version of the IASP91 model (in blue) and the PREM model (in magenta). (b) Zoom of (a) in the top 1,000 km of the mantle. (c) Zoom of (a) in the lowermost mantle.

Since SPECFEM3D_GLOBE has not been extensively tested at periods as short as *T*∼ 6 s, we first validate our calculations by reproducing some of the results of Komatitsch et al. (2010) using both SEM and GEM. Waveforms are calculated for a near‐vertical dip‐slip earthquake (strike = 0°, dip = 20°, rake = 45°) at the Earth's surface (depth = 0.1 km) at epicentral distances between 90° and 120°. As in Komatitsch et al. (2010), the seismic model is a simplified version of the IASP91 profile (Kennett & Engdahl, 1991; Figure 1). There is no shear attenuation, the crust is removed, and the discontinuities in the upper mantle have been replaced by strong gradients (Figure 1b).

**Figure 2:**
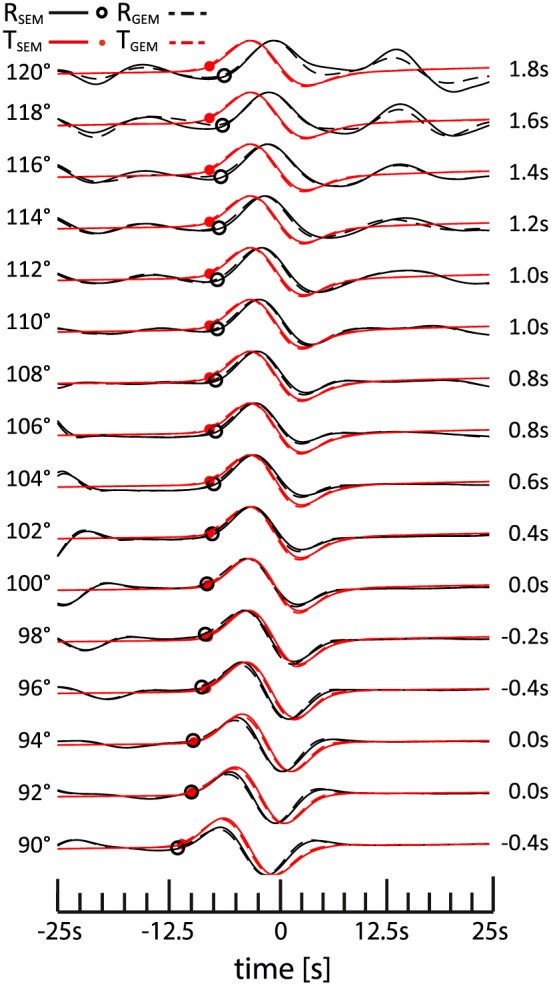
Comparisons between velocity waveforms calculated with spectral element method (SEM, solid lines) and seismograms using Gemini (GEM, dashed lines). The earthquake source is located at [latitude, longitude, depth] = [0°, 0°, 0.1 km] and has a focal mechanism with strike = 0°, dip = +20°, and rake = +45°. The seismic stations are placed on the equator to the east (at azimuth of 90°) at epicentral distances reported on the left of the waveforms. *S* *V* (black circles) and *S* *H* (red dots) onsets are marked on the waveforms. The apparent *S* *H*‐*S* *V* splitting is shown on the right of each pair of waveforms. Every waveform is normalized with respect to its own maximum amplitude. The timescale and reduction slowness (8.3 s/°) are as in Komatitsch et al. (2010)

The waveforms are convolved with a Gaussian source time function with a half‐duration of 6.5 s and filtered using a sixth‐order Butterworth band‐pass filter with corners at 7 and 80 s. Figure 2 replicates the results of Komatitsch et al. (2010) (their Figure 3). Our SEM and GEM simulations are equivalent. Minor differences in the radial components are visible at distances larger than 116° because *S* *V* amplitudes decrease strongly at distances larger than about 95° due to diffraction around the core. Even though the Earth model is isotropic, there is an apparent splitting between the SVdiff (on the radial component) and SHdiff waveforms (on the transverse component) that reaches 1.8 s at a distance of 120°. There are small differences in the splitting estimates between our and Komatitsch et al.'s (2010) study probably because of the slightly different waveform processing. However, the apparent splitting is confirmed.

### 3.2 Effects of Earthquake Source Depth

**Figure 3:**
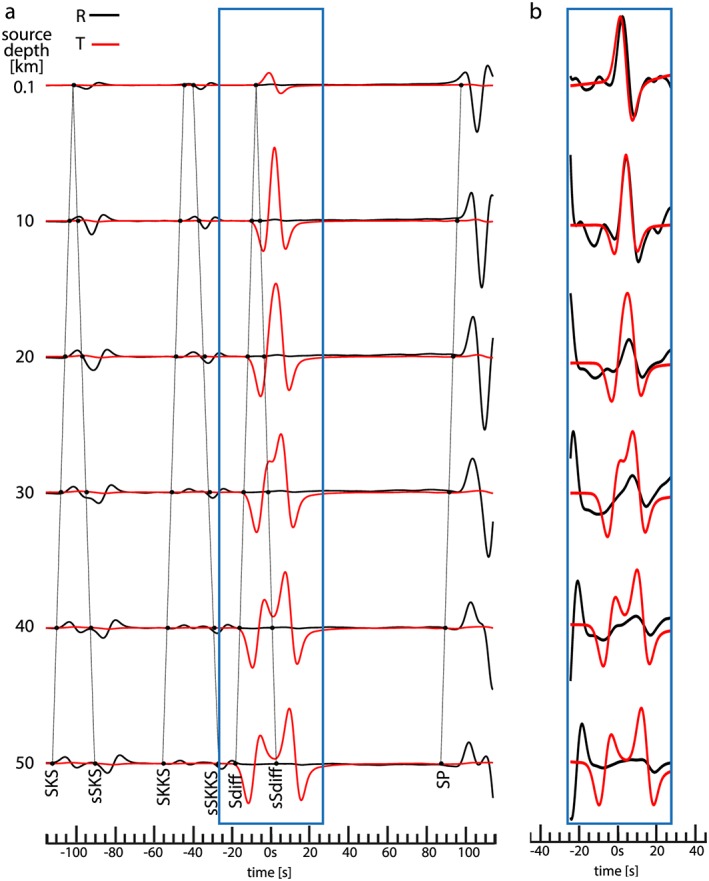
Effect of source depth on the differential arrival times of some seismic phases. (a) Velocity waveforms as in Figure 2 but for different source depths (reported on the left). The source mechanism and location are as in Figure 2, and all the waveforms are calculated at the epicentral distance of 110°. Each waveform is normalized with respect to its own maximum amplitude in each subplot. The timescale is the same for each subplot. Black lines show the theoretical onset arrival times for the main seismic phases. (b) Waveforms as in (a) but normalized in the time window included in the blue box (same time window as in Figure 2). The waveforms are all filtered with a sixth‐order Butterworth low‐pass filter with corner frequency 0.2 Hz.

To investigate the cause of the apparent splitting observed in the previous experiment, we show in Figure 3 waveforms at a distance of 110° for the same dip‐slip earthquake and the same source‐receiver azimuth as in Figure 2 but for focal depths of 0.1, 10, 20, 30, 40, and 50 km. The marked arrival times of several high‐amplitude phases are calculated using the TauP method (Crotwell et al., 1999) for the modified IASP91 model shown in Figure 1.

In Figures 2 and 3 (at depth 0.1 km), the apparent splitting seems to be related to Sdiff with positive polarity, both on the radial and transverse components. From the waveforms at depths larger than 30 km, it is evident that sSHdiff has a positive polarity and SHdiff has a negative polarity on the transverse component. At a depth of 0.1 km when SHdiff and sSHdiff arrive simultaneously, the sum of the two signals has a positive polarity because sSHdiff is stronger than SHdiff. Figure S3 (in the supporting information) illustrates in detail how SHdiff emerges from sSHdiff with a negative onset as the source depth increases from 1 to 5 km. The waveforms for source depths of 20 km and larger indicate that sSVdiff has a positive polarity and that SVdiff is very weak on the radial component. Therefore, the apparent splitting observed at a depth of 0.1 km (as in the example of Komatitsch et al., 2010) is due to a time shift between SHdiff+sSHdiff on the transverse component and sSVdiff on the radial component. The interference of Sdiff with sSdiff affects the radial and transverse components differently because sSVdiff is much weaker than sSHdiff. This suggests that the earthquake's focal mechanism can have a strong effect on the apparent splitting, which will be further investigated in future work. For completeness, Figure S4 (in the supporting information) presents results at an epicentral distance of 114°, for which the apparent splitting for a source at 0.1 km depth is larger than at 110° (Figure 2). Similar to Figure 3, once the negative polarity of SHdiff starts to emerge (in this case, for a source depth of  2 km), the splitting reduces, because the SHdiff and sSHdiff phases start to separate.

## 4 Apparent Splitting for a Deep Earthquake Source

**Figure 4:**
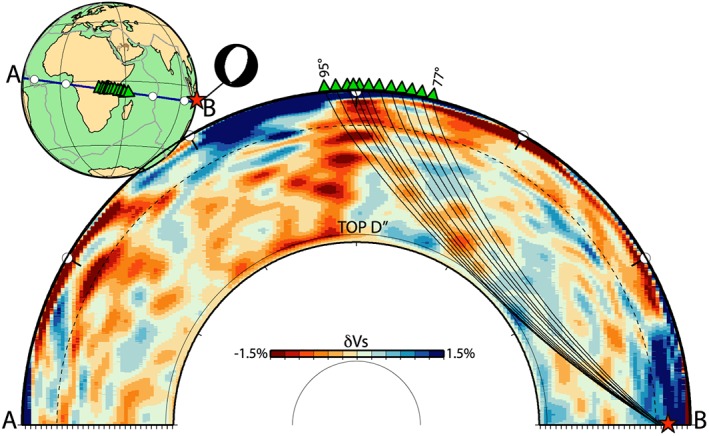
Source‐receiver configuration for the 30 August 1994 deep (604 km), *M*_*w*_ 5.8 Banda Sea earthquake. The source location is represented by a red star, and the focal mechanism is shown in the subplot on the top left of the figure. Receivers are represented by green triangles. The tomographic cross section shows the Vs perturbations of the isotropic part of the SGLOBE‐rani tomographic model with respect to the isotropic PREM model. Seismic rays connecting the source and receivers are calculated using PREM.

From here on, we compute seismic waveforms for deep earthquakes, which are typically used in shear wave splitting studies. Specifically, we use the source‐receiver path between the *M*_*w*_ 5.8, 30 August 1994 Banda Sea earthquake at a depth of 604 km (Figure 4) and stations in eastern Africa. For this normal faulting event, Ritsema (2000) measured *S* *H*‐*S* *V* splitting of 1–3 s (with *S* *H* faster than *S* *V*) at stations from a temporary network in Tanzania at epicentral distance of 87–91°.

### 4.1 Effects of 1‐D Velocity Structure in the D ${}^{\prime \prime }$ Layer

We systematically explore a range of isotropic models to investigate whether complexity in D ${}^{\prime \prime }$ can lead to apparent splitting in the same order of magnitude as reported for many high–velocity and low–velocity regions in the D ${}^{\prime \prime }$ layer. We assume the Preliminary Reference Earth Model (PREM) attenuation structure and source parameters from the global Centroid Moment Tensor (CMT) catalog (Dziewonski et al., 1981; Ekström et al., 2012). We convolve the synthetics with a Gaussian source time function with a half duration of 2.9 s (as reported in the GCMT catalogue) and apply the same band‐pass filter as before.

**Figure 5:**
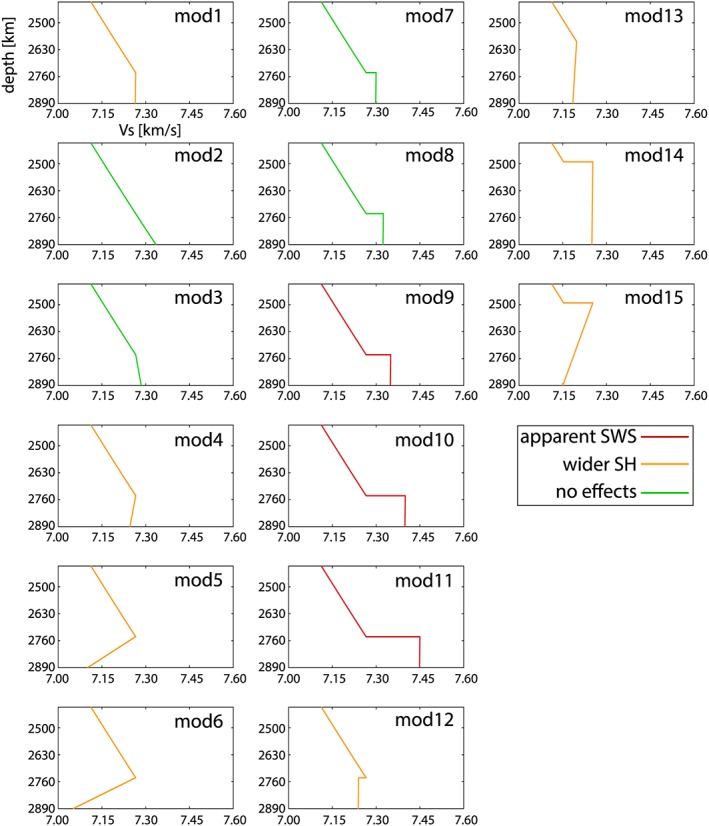
One‐dimensional isotropic models of the lowermost mantle used to simulate waveforms for the *M*_*w*_ 5.8 Banda Sea earthquake. Shallower parts of the models, not included in the plots, are as in PREM (see Figure 1). Color code of the velocity profiles is used to indicate cases in which either apparent splitting (red) or widening of the *S* *H* pulse (orange) or no effect on the waveforms (green) is observed in the corresponding theoretical waveforms.

Figure 5 shows the fifteen 1‐D isotropic Earth models for which we have synthesized waveforms. These models are based on the PREM model (mod1 in Figure 5), but the structure in D ${}^{\prime \prime }$ has been modified to represent the wide variety of shear velocity profiles previously proposed for different regions of D ${}^{\prime \prime }$. Models mod2–mod6 have different radial shear velocity gradients in D ${}^{\prime \prime }$ than in PREM. In mod2 the Vs gradient in D ${}^{\prime \prime }$ layer is constant throughout the lowermost mantle. The shear velocity gradients in models mod3 and mod4 are 1.27 × 10^−4^ and −1.33 × 10^−4^ s^−1^ in the lowermost 150 km of the mantle, respectively. The gradients in models mod5 and mod6 are −11 × 10^−4^ and 14 × 10^−4^ s^−1^, respectively. Similar negative gradients are observed in recent 3‐D global tomography models (e.g., Chang et al., 2015) and 1‐D profiles (Ritsema et al., 1997) across the LLVSPs. Models mod7–mod12 include velocity discontinuities at the top of D ${}^{\prime \prime }$. These velocity jumps range from 0.48% (in mod7) to 2.52% (in mod11). The strength of these velocity discontinuities is similar to that reported for downwelling regions (e.g., Helmberger et al., 2005; Sun et al., 2016; Yao et al., 2015; Young & Lay, 1987a, 1987b).

**Figure 6:**
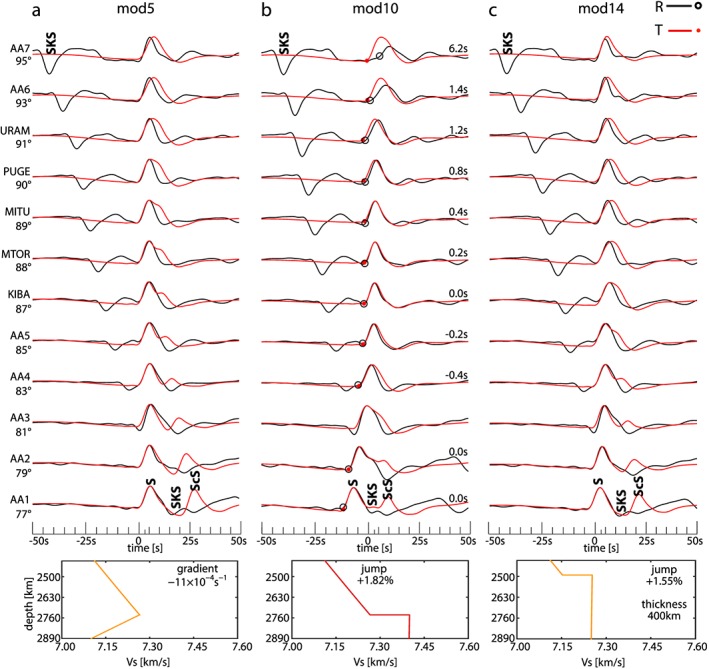
Examples of waveforms calculated for the *M*_*w*_ 5.8 Banda Sea earthquake. The source‐receiver geometry is shown in Figure 4. The 1‐D model used in the simulations is shown below each set of waveforms (see also Figure 5). On the left of the waveforms, the names of the stations and the epicentral distances are reported. Names of the seismic phases discussed in the text are reported. *S* *V* (black circles) and *S* *H* (red dots) onsets are marked on the waveforms. The apparent splitting is reported on the right of the waveforms. Every waveform is normalized with respect to its own maximum amplitude. (a) mod5, (b) mod10, and (c) mod14.

The waveforms are computed for stations from the Tanzania network and hypothetical stations along the source‐receiver great‐circle arc. As examples, waveforms for the models mod5, mod10, and mod14 are displayed in Figure 6. Figures S5–S7 (in the supporting information) show the waveforms calculated for all models in Figure 5.

We analyze the radial and transverse component waveforms computed for the 15 models in Figure 5 and classify the waveforms as having (i) no apparent *S* *H*‐*S* *V* splitting nor wider pulses (models labeled as “no effects” in green in Figure 5); (ii) *S* *H* pulses wider than *S* *V* (models labeled as “wider *S* *H*” in orange in Figure 5); and (iii) *S* *H*‐*S* *V* apparent splitting (models labeled as “apparent SWS” in red in Figure 5). We find that models mod9–mod11 with strong velocity discontinuities lead to a clear apparent splitting, while models mod4–mod6 and mod12–mod15, with low velocity in the lowermost mantle, cause a widening of the *S* *H* waveforms. The remaining models do not modify the *S* *H* and *S* *V* waveforms significantly.

For any realistic 1‐D reference model, *S* *c* *S* and *S* *K* *S* are the two high‐amplitude phases with similar arrival times to *S* between 77° and 95°. *S* *c* *S* arrives later than *S* and modifies the tail of the *S* wave at distances larger than about 80°. At distances shorter than about 81° *S* *K* *S* arrives earlier than *S* and can modify the *S* onset. The interference of *S* with *S* *c* *S* and *S* *K* *S* is different on the radial and transverse components. *S* *K* *S* is recorded only on the radial component and modifies the *S* *V* waveform only. *S* *c* *S* has the same polarity as *S* on the transverse component but opposite polarity on the radial component.

The interference of *S* with *S* *c* *S* depends also on the shear velocity structure. In the presence of a negative shear velocity gradient, *S* and *S* *c* *S* are more separated and the *S* *H* pulse is wider than in PREM. The *S* *H* pulse is particularly wide for models mod4–mod6 in Figure 5. Waveforms for mod5 (Figure 6a) show that the *S* *H* pulse widening is evident at distances from 90° to 95° (stations PUGE, URAM, AA6, and AA7). At distances shorter than 90° when *S* and *S* *c* *S* are separated by more than 4–5 s, the *S* *H* waveforms are double peaked (stations AA5, KIBA, MTOR, and MITU).

The interference of *S* with *S* *c* *S* for models with a shear velocity discontinuity at the top of D ${}^{\prime \prime }$ larger than 1.14% (models from mod9 to mod11) generates apparent splitting. The D ${}^{\prime \prime }$ discontinuity causes, in fact, an *S* wave triplication comprising a direct *S* wave (*S* *a* *b*), an *S* wave turning below the discontinuity (*S* *c* *d*), and an *S* wave reflecting off the discontinutiy (*S* *b* *c*). Their relative arrival times depend on the epicentral distance and velocity jump. For mod10 (Figure 6b), *S* *a* *b* arrives before *S* *c* *d* at distances shorter than 85° and the interference of the triplicated *S* and *S* *c* *S* results in a widening of *S* *H*. Between 85° and 87°, *S* *c* *d* arrives before *S* *a* *b* and the interference results in a negative apparent splitting. At epicentral distances larger than 88°, *S* *c* *S* arrives within the triplication and the interference results in a positive apparent splitting growing with the epicentral distance. At epicentral distances larger than 91°, *S* *b* *c* and *S* *a* *b* are no longer recorded. Although we measure large apparent splitting at these epicentral distances, the large difference in the waveforms prevent us from making further interpretations.

The range of epicentral distances where there is interference between the triplicated *S* and *S* *c* *S* depends nonlinearly on the depth and on the amplitude of the Vs jump at the top of the D ${}^{\prime \prime }$ layer. For example, despite the strong velocity jump, models mod14 and mod15 only produce a widening of the *S* *H* pulse—and no apparent splitting—in the range of epicentral distances analyzed (Figure 6c) because the layer is thick compared to models mod9–mod11.

**Figure 7:**
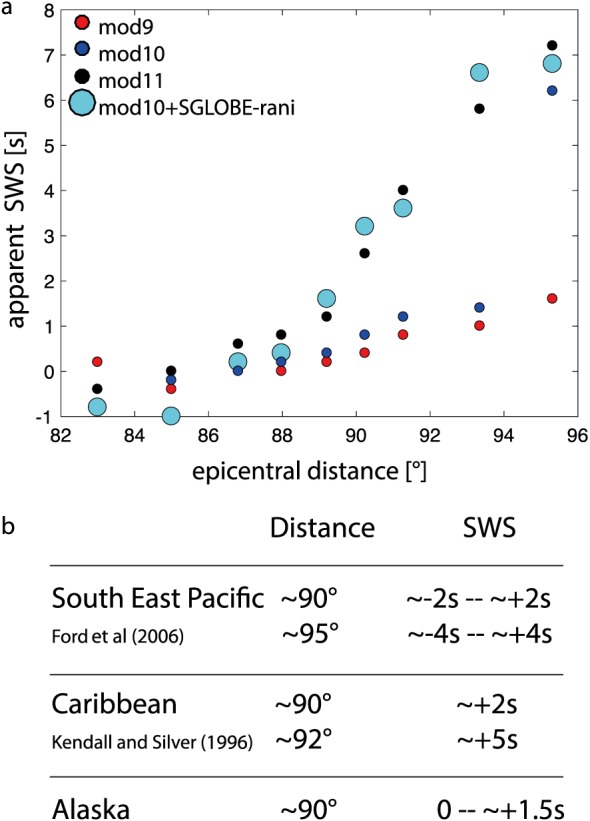
(a) Scatterplot of apparent shear wave splitting (SWS) values measured in this study against the epicentral distance. Small circles denote the 1‐D isotropic models with a Vs jump at the D ${}^{\prime \prime }$ discontinuity (mod9, mod10, and mod11, shown in Figure 5) for which apparent splitting is observed. Large circles denote the apparent splitting observed for the 3‐D model superimposing the isotropic part of SGLOBE‐rani on mod10. (b) Illustrative observed values of *S* *H*‐*S* *V* splitting from the literature for various D ${}^{\prime \prime }$ regions are reported for comparison.

The apparent shear wave splitting values measured for models mod9–mod11 are summarized in Figure 7a. We find small, negative shear wave splitting values (i.e., *S* *V* faster than *S* *H*) for most models of Figure 7a for the shortest (< 88°) epicentral distances. The largest, positive splitting values (up to ∼7.2 s) are seen at the largest distances. Overall, the range of shear wave splitting values measured in our synthetics is on the same order as measurements from real data reported in the literature (Figure 7b).

## 5 Effects of 3‐D Velocity Structure on the Apparent Splitting

To understand whether 3‐D velocity heterogeneity can complicate further the interpretation of shear wave splitting, we repeat some of the experiments described in sections 3 and 4 by incorporating 3‐D global tomographic models in the full waveform modeling simulations using SEM.

### 5.1 Deep Dip‐Slip Source Model

**Figure 8:**
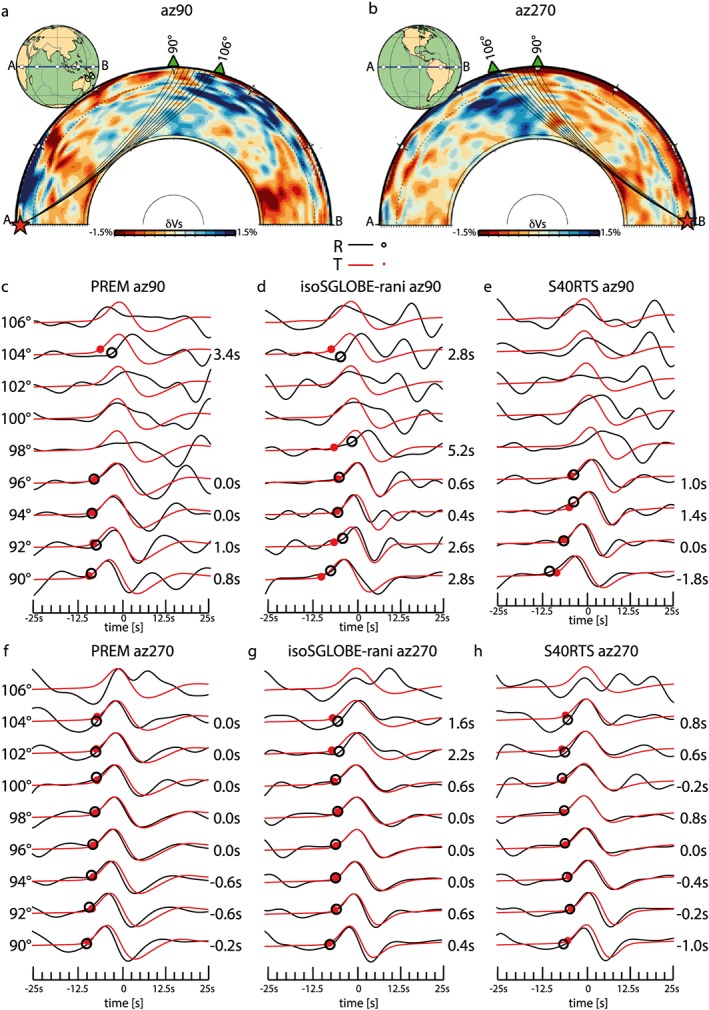
Effects of 3‐D Earth structure on the shape of *S* waveforms. (a) Rays in PREM are shown for *S* phases recorded from 90° to 106° for the same source as in Figure 2 but at source depth of 604 km. The stations are at an azimuth of 90°. The source location is represented by the red star, and the receivers are represented by green triangles. Vs perturbations of the isotropic part of the SGLOBE‐rani tomographic model with respect to the isotropic PREM are shown in the background of the cross sections. (b) As in (a) but for an azimuth of 270°. (c) Velocity waveforms calculated for the source‐receiver geometry in (a) and 1‐D Earth model PREM (isotropic). The epicentral distance range is shown on the left of the waveforms. *S* *H* (black circles) and *S* *V* (red dots) onsets are marked on the waveforms. The measured apparent splitting is indicated on the right. Every waveform is normalized with respect to its own maximum amplitude. (d) As in (b) but for the isotropic part of 3‐D Earth model SGLOBE‐rani. (e) As in (b) but for the 3‐D Earth model S40RTS. (f) As in (c) but for the geometry in (b). (g) As in (f) but for the isotropic part of 3‐D Earth model SGLOBE‐rani. (h) As in (f) but for the 3‐D Earth model S40RTS.

Figure 8 shows waveforms for the same dip‐slip source model used in section 3 but now for a source depth of 604 km, since shear wave splitting studies are typically based on deep earthquake data. We use two different 3‐D isotropic Earth models that include the global crustal model CRUST2.0 (Bassin et al., 2000), the PREM attenuation, and two whole mantle models: (i) S40RTS (Ritsema et al., 2011) and (ii) the isotropic part of the more recent SGLOBE‐rani model (Chang et al., 2015). Both 3‐D Earth models are defined as Vs perturbations with respect to the reference model PREM, and in our calculations we use an isotropic version of PREM (i.e., excluding PREM's upper mantle anisotropy). We also calculate reference waveforms for PREM to highlight the effects of the 3‐D Earth structure in the waveform analysis. Figure 8 shows waveforms simulated at azimuths of 90° and 270°.

Figures 8c and 8f show that the waveforms and the apparent splitting values obtained for PREM are different for the two azimuths. Apparent splitting ranges from 0.8 s to 3.4 s at an azimuth of 90° and from −0.6 s to −0.2 s at an azimuth of 270°. At an azimuth of 90°, a strong arrival on the radial component interferes with *S* *V* at a distance of 90° and moves out with distance. This signal arrives about 20 s after *S* at 96°. Travel‐time calculations show that this strong arrival is consistent with the arrival time of S^∧^220P, the *S* wave reflected off under the 220‐km mantle discontinuity. The interference between *S* and S^∧^220P has a minor effect on the waveforms at azimuth of 270° because of the higher amplitude ratio between *S* and S^∧^220P. Thus, the difference in waveforms and apparent splitting observed at the two azimuths is due to the focal mechanism used in this experiment that radiates seismic energy differently along the two azimuths analyzed.

For an azimuth of 90°, the *S* wave modeled in the 3‐D Earth models (Figures 8d and 8e) traverses the LLSVP beneath Africa just before traveling through the D ${}^{\prime \prime }$ region (Figure 8a). Moreover, the *S* wave traveling at distances larger than 102° crosses a high‐velocity anomaly in the uppermost ∼500 km of the mantle, before reaching the surface. Due to this shallow high‐velocity anomaly, the S^∧^220P arrives earlier than in the 1‐D model PREM. The different interference features in the two 3‐D Earth models cause different apparent splitting values.

For an azimuth of 270°, the *S* wave in the 3‐D Earth models (Figures 8g and 8h) crosses a low‐velocity mantle before traveling through D ${}^{\prime \prime }$. *S* waves traveling at distance larger than 104° also cross the high‐velocity anomaly of the South America slab between the D ${}^{\prime \prime }$ and the Earth's surface (Figure 8b). Waveforms and splitting for the two 3‐D Earth models are different from the corresponding ones calculated with the 1‐D model, and there are only two clear cases of observed splitting for the 3‐D mantle model SGLOBE‐rani (Figure 8g) at epicentral distances of 102° and 104°, where *S* is clearly diffracted. Borgeaud et al. (2016) attributed the Sdiff wave apparent splitting to the different sensitivity of *S* *V* and *S* *H* to the core‐mantle boundary (CMB). In our study, the differences in Vs structure near the CMB of the two 3‐D Earth models compared to the PREM model lead to distinct CMB conditions and thus possibly to the observed differences in waveforms and shear splitting values of the diffracted waves.

### 5.2 The 1994 *M*_*w*_ 5.8 Banda Sea Earthquake

In this section we use the same earthquake mechanism as in section 4 for the 30 August 1994 deep (604 km depth), *M*_*w*_ 5.8 Banda Sea earthquake. We superimpose the isotropic part of the global model SGLOBE‐rani (Chang et al., 2015) on the 1‐D model mod10 (see Figures 5 and 6), so that the 3‐D model includes a seismic velocity discontinuity at the top of D ${}^{\prime \prime }$. The mantle model is coupled with the global crustal model CRUST2.0 (Bassin et al., 2000) and PREM attenuation. The *S* wave crosses a succession of weak positive and negative velocity anomalies as it travels from the earthquake source to the D ${}^{\prime \prime }$ layer. On the other hand, from the D ${}^{\prime \prime }$ layer to the surface, *S* traverses an average slow region, notably for the longest paths (see Figure 4).

**Figure 9:**
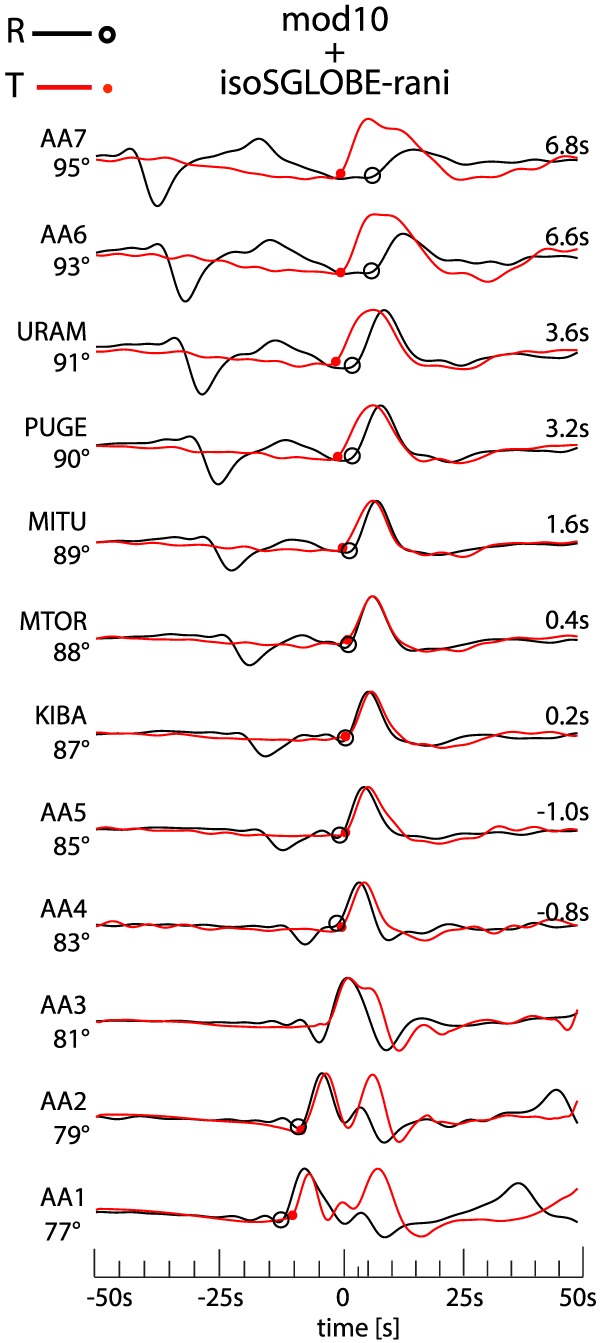
Effects of 3‐D Earth's structure on *S* waveforms. Waveforms calculated for the isotropic part of the SGLOBE‐rani model, superimposed to the 1‐D Earth model mod10. PREM's attenuation is included. On the left of the waveforms, the names of the stations and the epicentral distances are reported. *S* *V* (black circles) and *S* *H* (red dots) onsets are marked on the waveforms. The apparent splitting is reported on the right of the waveforms. The source‐receiver geometry is shown in Figure 4. Every waveform is normalized with respect to its own maximum amplitude.

Waveforms for this simulation are shown in Figure 9 together with the corresponding apparent splitting. The waveforms differ from the 1‐D simulation for mod10. The apparent splitting is as strong as that obtained for mod11 (see Figure 7a), which has a D ${}^{\prime \prime }$ discontinuity stronger than mod10. The 3‐D heterogeneity changes the *S* *c* *S* onsets compared to the 1‐D simulation and hence modifies its interference with the *S* phase. This can be seen in almost all the epicentral distances when comparing the waveforms for mod10 and for mod10+SGLOBE‐rani (Figures 6b and 9). Thus, the differences in apparent splitting between the 1‐D and 3‐D simulations are likely due to a distinct interference between the *S* *c* *S* and the triplicated *S* phase in the two types of simulations.

## 6 Discussion and Conclusions

Using 1‐D and 3‐D waveform simulations, we have demonstrated that phase interference can distort *S* *H* and *S* *V* waveforms and cause apparent splitting between *S* *H* and *S* *V* waveforms even in an isotropic Earth's model. The characteristics of interference and the magnitude of the shear wave splitting depend on the depth of the earthquake, seismic radiation pattern, D ${}^{\prime \prime }$ thickness, Vs discontinuities and gradients, and 3‐D Earth structure. The apparent splitting values obtained in this study are in the same order of magnitude as those reported in observational studies of shear wave splitting based on real data, which are often interpreted in terms of D ${}^{\prime \prime }$ anisotropy.

We have found that in most of the cases apparent splitting is due to the anomalous interference of the direct *S* phase with other seismic phases. For near‐surface earthquakes, notably for the source‐receiver configuration used by Komatitsch et al. (2010), and epicentral distances ranging from 102° to 120° the interference of Sdiff with sSdiff can produce splitting up to 1.8 s. For deeper earthquakes, when *S* does not interfere with *s* *S*, apparent splitting may be due to the interference of a triplicated *S* with *S* *c* *S* or of *S* with a precursor of *S* *P* due to an upper mantle reflection for a favorable radiation pattern. Strong negative Vs gradients in the D ${}^{\prime \prime }$ layer delay the onset of diffraction. Consequently, the separation of *S* and *S* *c* *S* broadens the *S* *H* waveform or produces a double‐peak shape at the shortest distances and a *S* *H* pulse wider than *S* *V* at the longest distances. If a strong discontinuity (> 1.14%) is located at the top of the D ${}^{\prime \prime }$, the interference of *S* triplicated at the discontinuity and *S* *c* *S* may lead to apparent splitting up to 7 s depending on the strength of the discontinuity and epicentral distance.

We also found that 3‐D Earth structure can modify the waveforms and enhance or reduce the apparent splitting. In fact, seismic heterogeneity affects not only the arrival time and waveform of the waves interfering with the direct *S* but also the epicentral distance at which *S* starts to diffract along the CMB.

Positive shear wave splitting (*V*_SH_>*V*_SV_) has been detected in several high D ${}^{\prime \prime }$ shear velocity regions underlying present or past subduction zones, such as beneath the Caribbean, Alaska, and North Siberia (e.g., Garnero & Lay, 1997; Kendall & Silver, 1996; Thomas & Kendall, 2002). Thus, many studies attribute it to positive D ${}^{\prime \prime }$ radial anisotropy due to slab deformation and/or the collision of slabs with the CMB. Slab deformation can produce laminated structures or lattice‐preferred orientation in constituent minerals, which could be compatible with radial anisotropy (e.g., McNamara et al., 2002). However, our results indicate that such geodynamic interpretations must be made cautiously, as other factors such as Vs discontinuities at the top of D ${}^{\prime \prime }$ can potentially produce similar apparent splitting.

This study follows previous studies that highlighted the possibility of apparent *S* splitting in D ${}^{\prime \prime }$. In early work, Maupin (1994) used approximate forward modeling schemes to show that the distinction between the effects of isotropic and anisotropic structure on the Sdiff waveforms is not trivial. Komatitsch et al. (2010) used the SEM to demonstrate that apparent splitting of Sdiff waves can occur for 1‐D Earth models. However, Komatitsch et al. (2010) considered an earthquake source very close to the surface and here we showed that the resulting apparent Sdiff splitting is due to interference of Sdiff and sSdiff for such a shallow source. Thus, in our simulations we also considered more realistic deep earthquake sources, which are typically used in real data studies to reduce such phase interference effects. Borgeaud et al. (2016) studied the apparent splitting of *S* due to finite frequency effects and attributed the Sdiff apparent splitting to the different sensitivity of *S* *V* and *S* *H* to the boundary conditions between the solid mantle and liquid outer core. In particular, they highlighted that apparent shear wave splitting can result from the misidentification of triplicated phases, which is compatible with our results. In addition, Kawai and Geller (2010) showed that the resolution of the velocity of *S* *V* shear waves very close to the CMB is inherently limited due to the boundary condition of zero tangential traction at the CMB. In this work we confirm the apparent splitting reported by these previous studies and we emphasize phase interference as being a key cause of apparent splitting. Moreover, our study also complements previous work by showing that 3‐D Earth structure can either enhance or reduce apparent splitting, depending on the region through which the waves propagate, which adds complexity to the shear wave splitting analysis. Nevertheless, we highlight that in the case of *S* waves diffracted along the core, phase interference and different sensitivity to the CMB can both cause apparent splitting.

In conclusion, we systematically quantified apparent shear wave splitting for several source depths and for a wide range of Earth models, including anomalous Vs gradients, D ${}^{\prime \prime }$ thickness, discontinuities, and 3‐D heterogeneity. Our analysis highlighted a strong interplay between the various source and structure parameters considered, which may lead to a misinterpretation of the splitting and potentially erroneous constraints on intrinsic D ${}^{\prime \prime }$ anisotropy. Full waveform modeling considering realistic sources and a wide range of 1‐D and 3‐D Earth models as in this study is a promising way to address these issues. While the illustrative examples based on the global smooth 3‐D Earth models used in this study are a useful first step to quantify their effect on apparent shear wave splitting, future efforts will be directed toward comprehensive 3‐D full wavefield analyses including more complex D ${}^{\prime \prime }$ structures and multiple source‐receiver orientations.

## Supporting information



Supporting Information S1
