# Supplementary material for: Apparent Splitting of S Waves Propagating Through an Isotropic Lowermost Mantle
Source: J Geophys Res Solid Earth. 2018 May 11;123(5):3909–22. doi: 10.1002/2017JB014394 (PMC6049884; doi:10.1002/2017JB014394)
Supplement: Supplementary file 1 — Supporting Information S1 [file JGRB-123-3909-s001.pdf]

JOURNAL OF GEOPHYSICAL RESEARCH

Supporting Information for "Apparent splitting of S waves propagating through an isotropic lowermost mantle"

L. Parisi,[1,2] A. M. G. Ferreira,[3,4] J. Ritsema,[5]

Contents of this file

---

[1]PSE Division, King Abdullah University of Science and Technology, Thuwal, Saudi Arabia

[2]School of Environmental Sciences, University of East Anglia, Norwich, UK.

[3]Department of Earth Sciences, University College of London, London, UK.

[4]CEris, ICIST, Instituto Superior Técnico, Universidade de Lisboa, 1049-001 Lisboa, Portugal.

[5]Department of Earth and Environmental Sciences, University of Michigan, Ann Arbor, Michigan, USA.

1. Figures S1 to S7

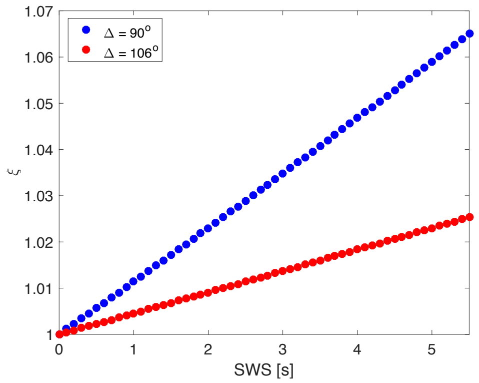

Figure S1. Radial anisotropy $\xi = V_{SH}^2/V_{SV}^2$ as a function of the shear wave splitting (SWS) between vertically (SV) and horizontally (SH) polarized shear waves for a homogeneous D" with 390-km thickness and Vs of 7.2 km/s.

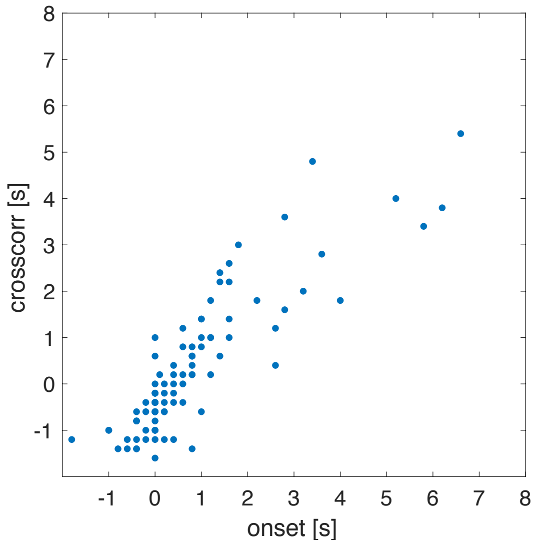

Figure S2. Scatter plot of the apparent splitting measured by cross-correlation (correlation coefficient > 0.85) and manual onset picking.

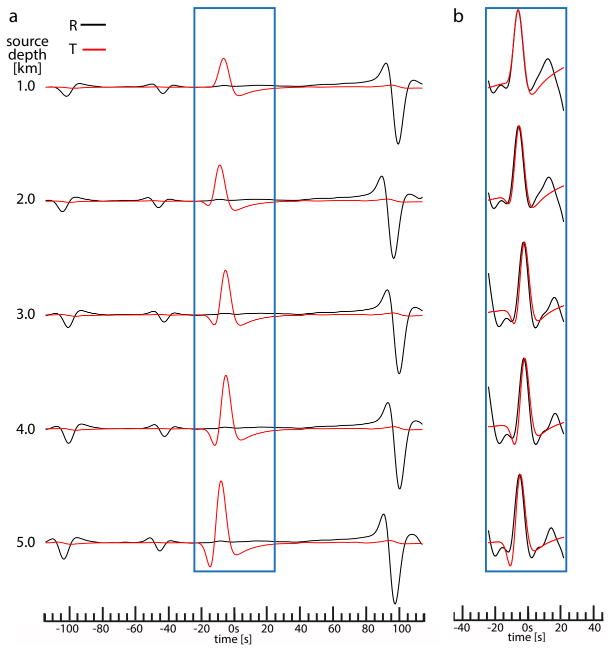

Figure S3. Effect of source depth on the differential arrival-times of body-waves. **a)** Velocity waveforms at the epicentral distance of 110°, as in Figure 3 but for different source depths (reported on the left). (b) Zoom of the waveforms in (a) around the Sdiff phase.

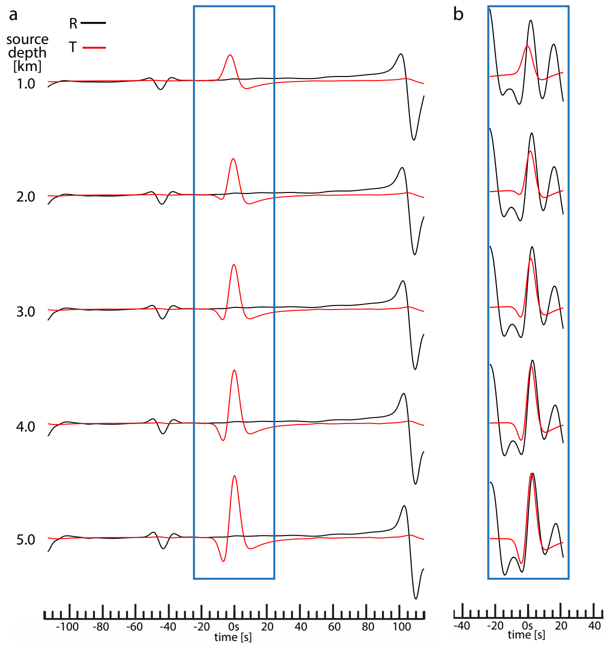

Figure S4. As in Figure S3 but for the epicentral distance of 114°.

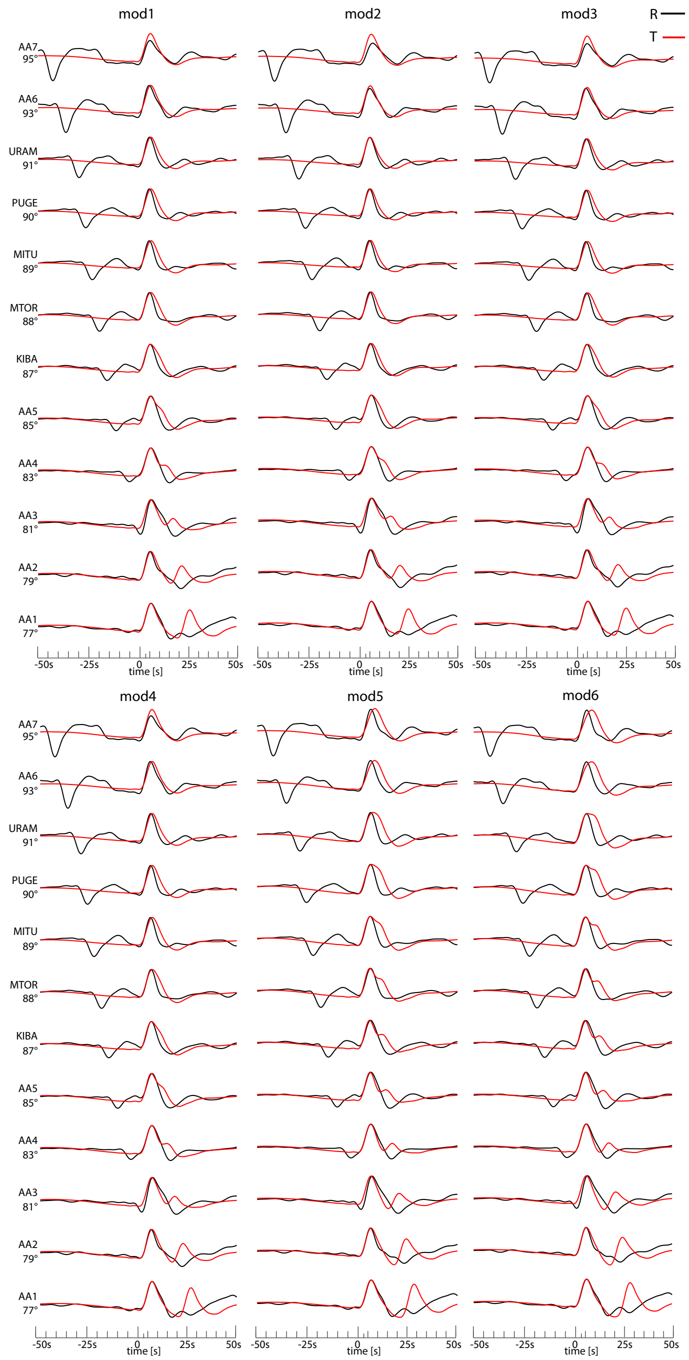

Figure S5. Examples of waveforms calculated for the Mw 5.8 Banda Sea earthquake. The source-receiver geometry is shown in Figure 4. The name of the 1-D model used in the simulation is reported at the top of each subplot and shown in Figure 5. On the left of the waveforms, the names of the stations and the epicentral distances are reported. Every waveform is normalized with respect to its own maximum amplitude.

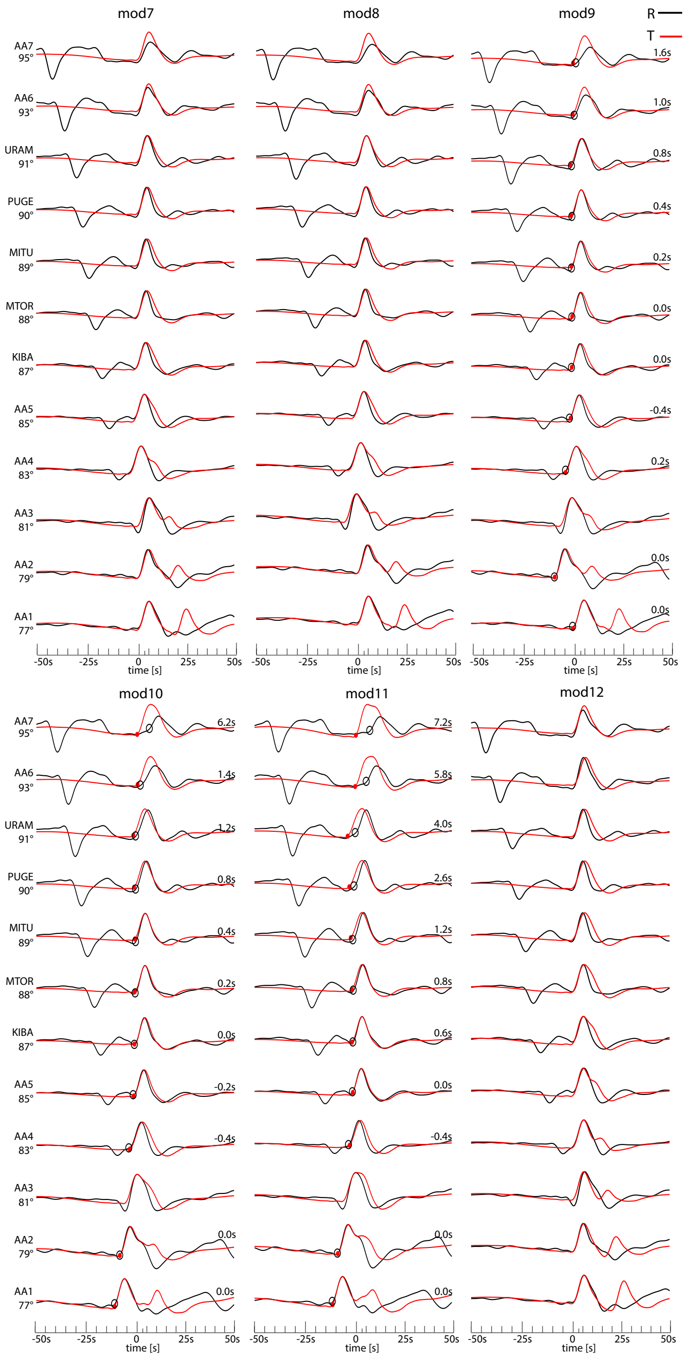

Figure S6. Same as in Figure S4, but for the 1-D Earth models mod7-mod12. SV (black circle) and SH (red dot) onsets are shown for mod9–mod11 for which apparent splitting is observed.

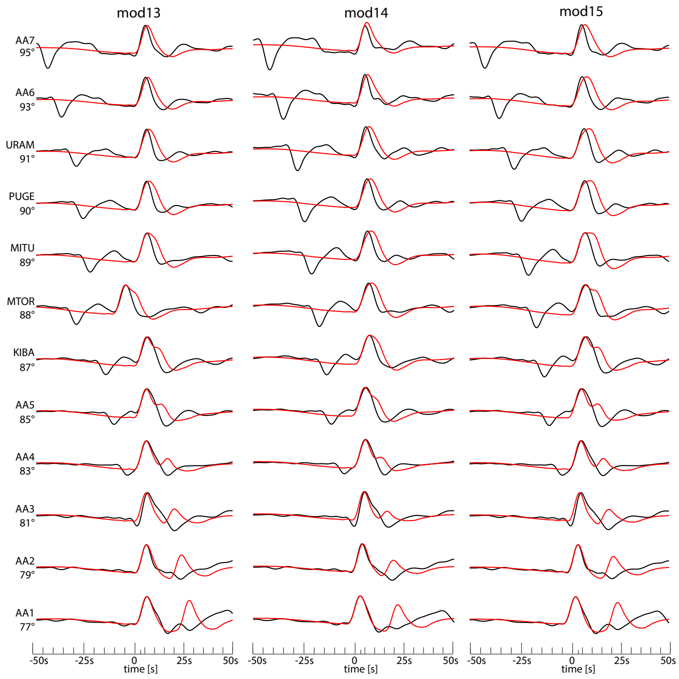

Figure S7. Same as in Figure S4, but for the 1-D Earth models mod13-mod15.
